# Anti-Fatigue Effect of *Prunus Mume* Vinegar in High-Intensity Exercised Rats

**DOI:** 10.3390/nu12051205

**Published:** 2020-04-25

**Authors:** Jeong-Ho Kim, Hyun-Dong Cho, Yeong-Seon Won, Seong-Min Hong, Kwang-Deog Moon, Kwon-Il Seo

**Affiliations:** 1 Department of Food Science and Technology, Kyungpook National University, Daegu 41566, Korea; kimjeoho90@gmail.com (J.-H.K.); kdmoon@knu.ac.kr (K.-D.M.); 2 Institute of Agricultural Life Sciences, Dong-A University, Busan 49315, Korea; chd0811@hanmail.net; 3 Department of Food Biotechnology, Dong-A University, Busan 49315, Korea; wonys@dau.ac.kr; 4 College of Pharmacy and Gachon Institute of Pharmaceutical Science, Gachon University, Incheon 21936, Korea; hongsm0517@gmail.com

**Keywords:** *prunus mume*, vinegar, anti-fatigue effect, high-intensity exercise, phenolic acid

## Abstract

Nowadays, new types of vinegar have been developed using various raw materials and biotechnological processes. The fruit of *Prunus mume* has been extensively distributed in East Asia and used as a folk medication for fatigue. In this study, the *Prunus mume* vinegar (PV) was produced by a two-step fermentation and was evaluated for its anti-fatigue activity by C2C12 myoblasts and high-intensity exercised rats. The administration of PV significantly improved running endurance and glycogen accumulation in the liver and muscle of PV supplemented rats compared to sedentary and exercised control groups. In addition, PV supplementation elicited lower fatigue-related serum biomarkers, for instance, ammonia, inorganic phosphate, and lactate. PV administered rats exhibited higher lactate dehydrogenase activity and glutathione peroxidase activity, and lower creatine kinase activity and malondialdehyde levels. Furthermore, phenolic compounds in PV were identified using HPLC analysis. The phenolic acids analyzed in PV were protocatechuic acid, syringic acid, chlorogenic acid, and its derivates. These results indicate that the administration of PV with antioxidative property contributes to the improvement of fatigue recovery in exhausted rats. The findings of this study suggest that the PV containing various bioactive constituents can be used as a functional material against fatigue caused by high-intensity exercise.

## 1. Introduction

*Prunus mume* Sieb. et Zucc., which is known as maesil, ume, and meizi, is widely cultivated in Korea, Japan, and China and has been used for a long time as a folk remedy for digestion, thirst, detoxification, vomiting and fever [1]. Previous studies on the pharmacological and biological activities of maesil have investigated it as a potential source of free radical scavengers, as an inhibitor of the influenza A virus and the motility of *Helicobacter pylori,* and as a pro-inflammatory mediator, as well as its ability to improve blood fluidity [1,2,3]. In addition, maesil extract has been shown to exert anti-fatigue activities in trained rats [4]. Although there are a number of studies using maesil extract, studies on processed food using maesil have not fully been explored. Hence, this study aimed to develop a vinegar using maesil and investigate its anti-fatigue activity.

Vinegar is an alkaline product that has long been used as a relish and traditional medication [5]. Recently, many sorts of vinegar have been developed using fundamental sources and technologies to satisfy customer needs. Because the major components of vinegar have shown numerous beneficial effects, e.g., antioxidant, anti-hypertensive, anti-hyperglycemic and antimicrobial effects, it is popularly consumed worldwide [6,7,8,9]. Furthermore, previous studies have shown that the administration of acetic acid elevates glycogen repletion in the liver and skeletal muscles of exhausted rats during exercise and that orally supplemented acetate induces muscle glycogen synthesis after intensive exercise in horses [10,11]. These studies have suggested that continuously supplemented vinegar elicits valuable effects on endurance exercise capacity and recovery from physical fatigue. However, the physiological changes underlying the anti-fatigue effects of maesil vinegar are not yet fully understood.

Fatigue, a common symptom in most communities that many people have experienced, is considered as difficulty in starting or maintaining spontaneous activities, and deterioration of exercise performance [12]. Many studies have shown that various factors are important when considering fatigue and exercise. For example, high-intensity exercise-induced exhaustion is related to fatigue which indicates that the working muscle capacity is severely damaged [13]. In addition, high-intensity exercise induces the reduction of energy sources, e.g., liver and muscle glycogen, as well as the accumulation of metabolites, including lactic acid, inorganic phosphorus and ammonia which induce muscle fatigue by intracellular acidosis in the body [12,14]. Thus, recovery from exercise-induced fatigue requires that bodily damage has been repaired and that metabolites accumulated during exercise be eliminated. In addition, oxidative stress has been reported to cause various chronic diseases, e.g., chronic fatigue, skin aging, diabetes mellitus, cancers, and Alzheimer’s disease [15,16,17,18]. For these reasons, researchers have investigated natural products for their ability to improve physical capabilities, such as the reduction of fatigue and increased exercise endurance with few side effects. 

Therefore, in the present study, vinegar containing a high level of organic acids and amino acids was produced via two-step fermentation using maesil supplemented with pear juice as a substrate. The anti-fatigue activities of *Prunus* mume vinegar (PV) were then estimated based on the effects of cell viability and glycogen accumulation *in vitro* and the changes of fatigue-related biomarkers *in vivo*.

## 2. Materials and Methods

### 2.1. Materials

*Prunus mume* juice (PJ) was produced by the method of Cho et al. [19]. *P. mume* fruits (maesil) were obtained from the Korea Maesil Organization (Suncheon, Korea). Maesil were sorted, meticulously washed with water, crushed then reacted with 0.1% (w/v) pectinase (Pectinex Ultra AFP, Novozyme, Switzerland, 10,000 Pectu/g) to disrupt cellular wall at 40 °C for 2 hours. Next, the reacted maesil were centrifuged at 3500× *g* for 15 min at 4 °C. The supernatant was filtered using filter paper (Whatman No.2, 8 μm) and concentrated by rotary evaporator at 30 °C until 56–60°Brix was reached. The pear extract was obtained from ESfood Co. (Gunpo, Korea), then kept at 4 °C to preserve its qualities. Its characteristics were as follows: 69°Brix, pH 3.4–3.6, and 0.52–0.61% acidity. *Saccharomyces cerevisiae* KCCM 11306 and *Acetobacter aceti* KCCM 12654 were obtained from the Korea Culture Center of Microorganisms (Seoul, Korea).

### 2.2. Production of PV

Alcohol and acetic acid fermentation using the PJ were performed three times by batch culture. Before starting the alcohol fermentation, 30 mL of PJ and 750 mL of distilled water were mixed and fortified with pear extract to 16°brix as an initial broth. In the alcohol fermentation step, *Saccharomyces cerevisiae* KCCM 11306 (5%, v/v) was inoculated to 3% (v/v) PJ as a starter, after which it was cultivated in an incubator at 30 °C for 2 days. At the end of the alcohol fermentation, the *Prunus mume* wine (PW) was filtered through 110 mm pore-size filter paper and developed in a shaking incubator with *Acetobacter aceti* KCCM 12654 (10%, v/v) at 30 °C and 200 rpm for 10 days. To remove *Acetobacter*, PV was centrifuged at 1700× *g* for 5 min, after which the supernatants were separated. As a result, PV with a total acidity of 5.7% was developed and stored at 4 °C (Figure S1).

### 2.3. Physicochemical Properties of PV

#### 2.3.1. Total Acidity, Alcohol Content and Sugar Content in PV

The alcohol content of PV was measured by a Gay-Lussac hydrometer. Briefly, 100 mL of PV was taken from the flask and centrifuged for 10 min at 1800× *g* to get rid of *Saccharomyces cerevisiae* KCCM 11306. Next, the supernatant was distilled and readjusted to 100 mL with distilled water. The temperature of the distillate was then cooled until it reached 15 °C, after which the alcohol content was determined using an alcohol hydrometer. The sugar content of PV was measured using a hand-held refractometer (Atago pocket PAL-3, Atago Co., Fukaya, Saitama, Japan). Finally, the total acidity of PV was analyzed by titrating the diluted sample with 0.1 N NaOH until pH 8.3 and expressed as a quantity of acetic acid.

#### 2.3.2. Organic Acids and Free Amino Acids Content in PV

The organic acid composition was determined by high-performance liquid chromatography (Shimadzu Co. Model Prominence, Kyoto, Japan). The separation of organic acids was accomplished using a PL Hi-Plex H column (7.7 × 300 mm, Agilent Co., Santa Clara, CA, USA) at 65 °C. The mobile phase consisted of 5 mM H_2_SO_4_ and the flow rate was kept constant at 0.6 mL/min. The chromatographic peak coinciding with each organic acid was identified by comparing the retention time with that of each standard.

The free amino acid contents were analyzed using an amino acid autoanalyzer (L-8900, Hitachi, Tokyo, Japan) with an ion exchange column packed with Hitachi custom ion exchange resin (2622 SC PF, 4.6×60 mm). The column was maintained at 50 °C in a column oven and the temperature of the reactor was 135 °C. For the mobile phase, a buffer set (PF-1, PF-2, PF-3, PF-4, PF-6, PF-RG, R-3 and C1, Kanto Co., Tokyo, Japan) was used with a flow rate of 1 mL/min. Each free amino acid was identified by comparing the retention time with that of amino acids mixture standard solution type AN-Ⅱand B (FUJIFILM Wako Pure Chemical Co., Osaka, Japan).

### 2.4. Cytotoxicity and Glycogen Accumulation in Vitro

#### 2.4.1. Cell Culture and Differentiation

The C2C12 cells (mouse myoblasts) was purchased from American Type Culture Collection (ATCC, Rockville, ND, USA). The cells were cultured in Dulbecco’s Modified Eagle’s Medium (DMEM) supplemented with 10% fetal bovine serum (FBS), penicillin (100 IU/mL), and streptomycin (100 μg/mL) (Gibco, Life Technologies, Grand Island, NY, USA). The C2C12 cells were incubated at 37 °C under a humidified atmosphere with 5% of CO_2_ condition. To induce differentiation, 70% confluent cells were then cultured in DMEM supplemented with 2% horse serum (HS) and 10 μg/mL of insulin for 3 days with medium changes every two days.

#### 2.4.2. Sulforhodamine B (SRB) Assay

The cell proliferation was evaluated by sulforhodamine B (SRB, Sigma, St. Louis, MO, USA) assay. The C2C12 cells were seeded at 1 × 10^4^ cells/well in 48-well plates and differentiated by switching medium. The cells were then incubated with 0.1–0.4 μg/mL of PV for 3 days at 37 °C in a 5% CO_2_ humidified incubator. After treatment, medium was discarded and the cells were stained with SRB solution at room temperature for 1 hour, and washed five times using 1% acetic acid. Each well was solubilized with 10 mM Tris and measured at 540 nm by a microplate reader (Molecular Devices, Inc., San Jose, CA, USA)

#### 2.4.3. In Vitro Glycogen Content

To evaluate the effect of the samples on glycogen accumulation in C2C12 myoblasts, the glycogen content in the cells was determined using glycogen assay kit (Cell Biolabs, Inc., San Diego, CA, USA). Amyloglucosidase hydrolyzes glycogen to glucose and the glucose is oxidized by glucose oxidase, which produces hydrogen peroxide. The hydrogen peroxide is detected with a colorimetric probe. The color was measured at 540 nm using a microplate reader and glycogen content was calculated using a standard curve.

### 2.5. Animal Experimental Design

#### 2.5.1. Animals and Diets

Four-week-old male Sprague-Dawley (SD) rats were purchased from Hyo-Chang Science Inc. (Busan, Korea). The rats were individually divided into acryl cages and housed at 22 ± 2 °C on a 12 h light-dark cycle. All rats were fed pellets of commercial chow for the experimental period. The rats were then randomly divided into five groups (*n* = 6): sedentary control (SC), exercised control (EC) and exercised rats administered with 3% condensed *Prunus mume* juice (PJ), 5% PV diluted with distilled water (PV5) and 7.5% PV diluted with distilled water (PV7.5). All groups were supplemented by oral administration at a concentration of 7 mL/kg body weight for the experimental period, considered the daily intake volume in humans. Supplementation of a high concentration acetic acid is able to cause intestinal inflammation in rats. The PV7.5 was used as a high concentration for the experiment [12]. SC and EC rats were administered with equal amounts of distilled water. Afterward, all rats were elicited to run on a treadmill. During the experiment, rats had free access to food and water until the last 12 h of the experimental period, at which time food was withheld. All rats were treated in strict accordance with the Dong-A University guidelines for the care and use of laboratory animals (DIACUC-17-1).

#### 2.5.2. Gradual Loaded Exercise Program and Running Endurance Test

All rats excluding the SC group were trained through the gradual load exercise program from 09:00 to 13:00, 6 days per week for 4 weeks using a treadmill (Daejong Instrument Industry, Seoul, Korea). The program involves a gradually increased intensity with running at 20 m/min for 10 min, 25 m/min for 20 min, 30 m/min for 20 min and 35 m/min for 30 min from weeks 1 to 4, respectively. When rats are exhausted and unable to run, an electric shock board at the end of the treadmill regulated them to keep running.

At the end of the experimental period, rats (*n* = 6) were forced to run at 40 m/min until exhausted, and their running records were noted to determine running endurance. All rats were assessed as exhausted when they stayed on the electric board for over 10 s. The others (*n* = 6) were placed on the treadmill with a speed of 40 m/min for 60 min. After the experiment, rats were sacrificed with ethyl ether and blood samples were gathered from the inferior vena cava and placed at room temperature for 2 h, and then centrifuged at 2500× *g* for 20 min to separate the serum samples. The liver and gastrocnemius muscle were collected and rinsed with saline. All samples were stored at −80 °C in a deep freezer.

### 2.6. Biochemical Parameters

#### 2.6.1. Biomarkers Related to Fatigue

The levels of serum inorganic phosphate and ammonia were evaluated using Biovision Inc. (Milpitas, CA, USA). Lactate levels in the serum were determined using a lactate assay kit (Bioassay Systems, Hayward, CA, USA).

#### 2.6.2. Analysis of Glycogen Levels in Liver and Muscle

Glycogen content was analyzed according to the method described by Cho et al. [5]. Briefly, 0.2 g of tissues from liver and muscle were reacted with 400 μL of 30% potassium hydroxide solution, boiled for 30 min and then cooled at 25 °C. Next, 1 mL of ethanol was added to the mixture and it was centrifuged at 6000× *g* and 4 °C for 15 min. The supernatant was subsequently removed and the pellet was mixed with 0.5 mL of distilled water, after which 0.2% of anthrone solution was added to hydrolyze the glucose. Finally, the absorbance was measured at 620 nm with a spectrophotometer.

#### 2.6.3. Activities of Muscle Lactate Dehydrogenase (LDH) and Serum Creatine Kinase (CK) Activities

To evaluate muscle biomarkers related to lactate metabolism, gastrocnemius was prepared from the hindlimbs of rats. Briefly, 5 ml of 100 mM potassium phosphate buffer was added to 0.1 g of muscle tissue, after which samples were homogenized. The homogenates were then centrifuged at 10,000× *g* at 4 °C for 15 min and the supernatants were used for analysis. The activities of LDH and CK were determined by colorimetric kits (Bioassay Systems, Hayward, CA, USA).

#### 2.6.4. Levels of Malondialdehyde (MDA) and Glutathione Peroxidase (GPx) Activity

The MDA level and GPx activity were evaluated by homogenizing aliquots of 0.1 g of frozen liver in phosphate buffered saline (PBS). Following homogenization, samples were centrifuged at 3500× *g* at 4 °C for 10 min, then the supernatants were used for analysis. The MDA level and GPx activity were measured with colorimetric kits (Biovision Inc., Milpitas, CA, USA).

### 2.7. Determination of the Total Phenolic Content (TPC)

TPC of PV was determined by the Folin–Ciocalteu colorimetric method with some modifications [19]. Briefly, PV was reacted with Folin-Ciocalteu reagent and neutralized with sodium carbonate solution. Then, the absorbance of the blue color was measured at 760 nm by spectrophotometer. As the standard, gallic acid (Sigma-Aldrich, purity > 99%) was used and the TPC was expressed as mg gallic acid equivalents/g (mg GAE/g) PV.

### 2.8. HPLC Analysis

In the sample preparation for HPLC analysis, 10 mL of PV was filtered with 0.2 μm PVDF syringe filter (Adventec, Tokyo, Japan) and concentrated at 37 °C by rotary evaporator (EYELA, Tokyo, Japan). The PV concentrates were then diluted with distilled water at 50 mg/mL. The contents of phenolic compounds in PV were identified by HPLC-PDA (Shimadzu Inc., Walnet Creek, CA, USA) comprising a solvent delivery unit LC-20A, an auto-sampler SIL-20A, a photodiode array detector SPD-M20A, and a UV-VIS detector SPD-20A. The column temperature was kept at 40 °C in a CTO-20A column oven. After injecting 10 μL of sample, separation was performed in a Phenomenex C18 column (5 μm, 250 mm × 4.6 mm I. D.). For detection and quantification of compounds, chromatograms were recorded at 205, 210, 216.8 and 324.9 nm in the photo-diode detector. The separation process was carried out using a ternary mobile phase gradient consisted of 0.1% trifluoroacetic acid in water (solvent A), and acetonitrile (solvent B) at a flow rate of 1 mL/min. The percentage composition of solvent A was maintained at 92% for 10 min, 80% for 50 min, and then gradually decreased to 0% for 15 min, and finally increased to 95% for 20 min. Phenolic compounds, protocatechuic acid, syringic acid, chlorogenic acid, neochlorogenic acid and cryptochlorogenic acid were eluted at retention time ranges of 8.738, 23.784, 18.663, 9.660 and 20.395 min, respectively. All standard compounds were purchased from Chengdu Must Bio-Technology Co., Ltd. (ChengDu, China, purity > 98%).

### 2.9. Statistical Analysis

All data are presented as the means ± S.E. Data were evaluated by one-way analysis of variance using the SPSS (Chicago, IL, USA) software and by determining differences among the means using Duncan’s multiple-range test. Values were considered statistically significant at *p* < 0.05. 

## 3. Results and Discussion

### 3.1. Contents of Organic Acids and Free Amino Acids in PV

The major taste compounds in fermented vinegar consist of organic acids generated from fermentation, as well as free amino acids produced by hydrolyzation of protein during fermentation [20]. PV contained organic acids, acetic acid, oxalic acid, citric acid, succinic acid, malic acid and lactic acid, at 4034.46, 72.76, 1530.65, 1075.51, 140.95 and 390.87 mg%, respectively (Table 1). In addition, PV contained a number of free amino acids, namely, aspartic acid, tyrosine, phenylalanine, histidine, lysine and arginine. The contents of aspartic acid, tyrosine, phenylalanine, histidine, lysine and arginine were 7.56, 5.46, 4.43, 32.93, 4.11 and 20.76 ppm, respectively. After a two-step fermentation, PV showed higher organic acids, especially acetic acid, and free amino acid contents than PJ. Compared with previous studies, the contents of organic acids in commercial vinegar with sorghum consisted of acetic acid (3600 mg%), oxalic acid (16.62 mg%), citric acid (49.7 mg%), succinic acid (92.5 mg%), malic acid (27.83 mg%) and lactic acid (820 mg%) [21]. PV contained lower amounts of free amino acids than garlic vinegar, which contained high amounts of the free amino acids (23.4 ppm), tyrosine (not detected), phenylalanine (313.9 ppm), histidine (4.6 ppm), lysine (460.3 ppm) and arginine (65.0 ppm). Na et al. (2013) reported that the quality characteristics in fermented vinegars depend on different ingredients and are related to the higher amount of citric acid, succinic acid, malic acid, tyrosine and histidine, and lower amount of aspartic acid, phenylalanine, lysine and arginine, which were observed in PV compared with other fermented vinegars [22]. Overall, the results indicate that PV fermented with PJ fortified with pear extract contains a high amount of organic acids and various free amino acid contents.

### 3.2. Identification and Quantification of Phenolic Compounds in PV

**Figure 1:**
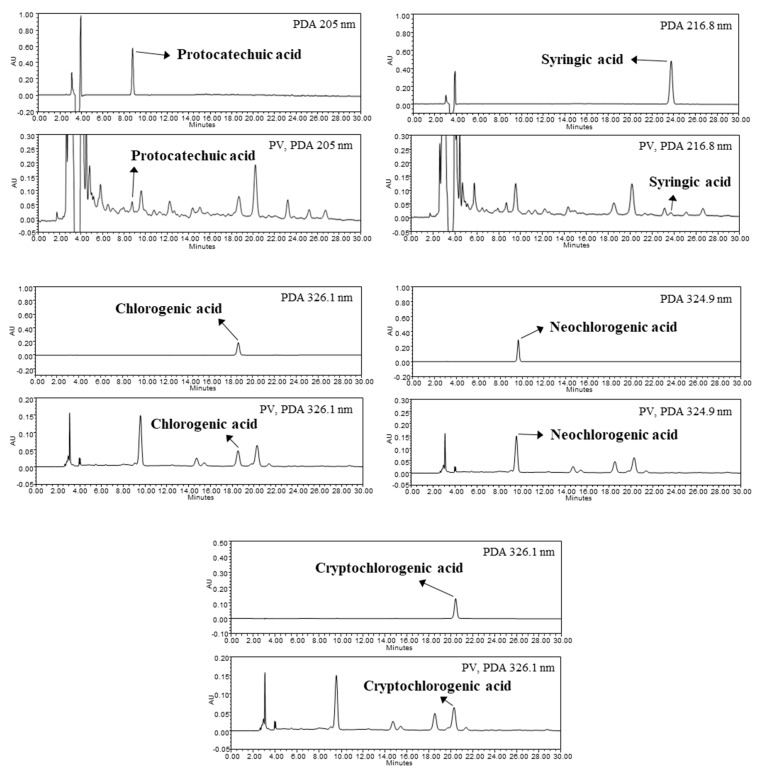
Phenolic compounds in *Prunus mume* vinegar (PV) analyzed by HPLC. Protocatechuic acid (205 nm, 8.774 min); syringic acid (216.8 nm, 23.857 min); chlorogenic acid (326.1 nm, 18.663 min); neochlorogenic acid (324.9 nm, 9.660 min); cryptochlorogenic acid (326.1 nm, 20.395 min).

The TPC of PV was 25.86 mg GAE/g (data not shown). To further identify the phenolic compounds present in PV, HPLC-PDA analysis was performed. Protocatechuic acid, syringic acid, chlorogenic acid, neochlorogenic acid and cryptochlorogenic acid with concentrations of 0.08, 0.22, 0.37, 0.82 and 1.36 mg/g, respectively, were identified using HPLC analysis by comparison with each standard phenolic acid (Figure 1). This result indicated that cryptochlorogenic acid and neochlorogenic acid were the main phenolic acids in PV. Many studies have reported that phenolic compounds affect functional properties, such as antioxidant, anti-cancer and anti-diabetes [23,24,25]. In a related study done by Yuan et al. (2019), polyphenol-rich *Sonchus arvensis* extract containing chlorogenic acid, luteolin and chicoric acid improved antioxidant enzyme activities and glycogen synthesis in exercise trained mice [26]. The aqueous extract of *Abelmoschus esculentus* Moench seeds containing high amounts of polyphenols and flavonoids showed significant antioxidant and anti-fatigue effects in mice after weight-loaded swimming test [27]. Also, phenolic compounds including 5-HMF, neochlorogenic acid, protocatechuic acid and syringic acid were identified in pectinase treated *Prunus mume* fruit concentrate which have displayed inhibitory effects on colorectal cancer cells [19]. Although further studies are required to investigate the molecular mechanisms behind the anti-fatigue activities of the phenolic compounds, this result indicates that the anti-fatigue activities of PV were related to its phenolic compounds, such as protocatechuic acid, syringic acid, chlorogenic acid and its derivates.

### 3.3. Effects of PV on Cell Proliferation and Glycogen Accumulation in C2C12 Myoblasts

**Figure 2:**
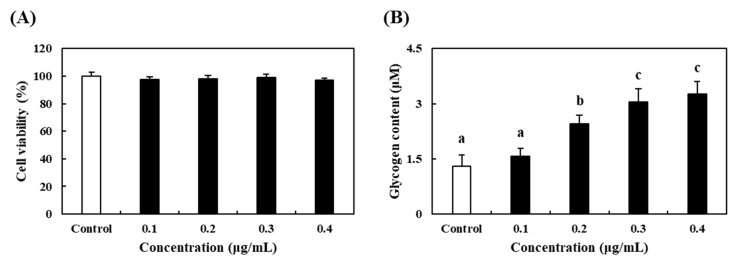
Effects of PV on (**A**) cell proliferation and (**B**) glycogen accumulation in C2C12 myoblasts. Data values are expressed as the means ± S.E. (*n* = 3). Different letters on the bar are significantly different (*p* < 0.05).

The skeletal muscle plays an important role supporting energy production in the body [27]. As shown in Figure 2, we evaluated cytotoxicity and glycogen accumulation of PV on C2C12 myoblast. To evaluate the cytotoxicity of PV, SRB assays were conducted in C2C12 myoblasts, and after differentiation the cells were treated with various concentrations of PV (0.1, 0.2, 0.3 and 0.4 μg/mL) for 48 hours (Figure 2A). The cell viabilities of C2C12 myoblasts treated with PV were over 95%, signifying no significant differences compared to control. To assess the glycogen content in C2C12 myoblasts, glycogen assay using cell lysates was carried out. As shown in Figure 2B, the glycogen contents in C2C12 myoblasts were significantly increased by PV in a dose-dependent manner. However, the treatment of PV at a dose of 0.4 μg/mL did not show significant differences compared to 0.3 μg/mL of PV. These results suggested that PV treatment can enhance glycogen accumulation with non-cytotoxic concentrations in skeletal muscle.

### 3.4. Effects of PV on Treadmill Running Time

**Figure 3:**
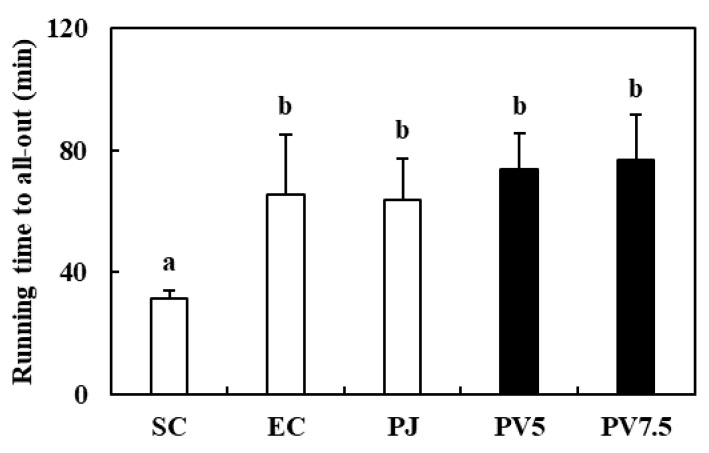
Effect of PV on running endurance time. Data values are expressed as the means ± S.E. (*n* = 6). SC: sedentary control, EC: exercised control, PJ: *Prunus mume* juice, PV5: 5% *Prunus mume* vinegar drink, PV7.5: 7.5% *Prunus mume* vinegar drink. Different letters on the bar are significantly different (*p* < 0.05).

The running time until exhausted is a marker of exercise capacity that represents fatigue recovery [5]. In this study, an exercise training program using treadmill was conducted to rats for 4 weeks. After high-intensity exercise to exhaustion, all groups revealed significantly increased running endurance compared with the SC rats, and PV7.5 recorded the longest running time among all groups (Figure 3). Reidy & Rasmussen (2016) reported that supplementation with amino acids increased exercise performance via induction of protein synthesis in human skeletal muscle after resistance exercise [28]. This result indicated that PV effectively increased endurance capacity in high-intensity exercised rats.

### 3.5. Effects of PV on Serum Biomarkers Related to Fatigue

**Figure 4:**
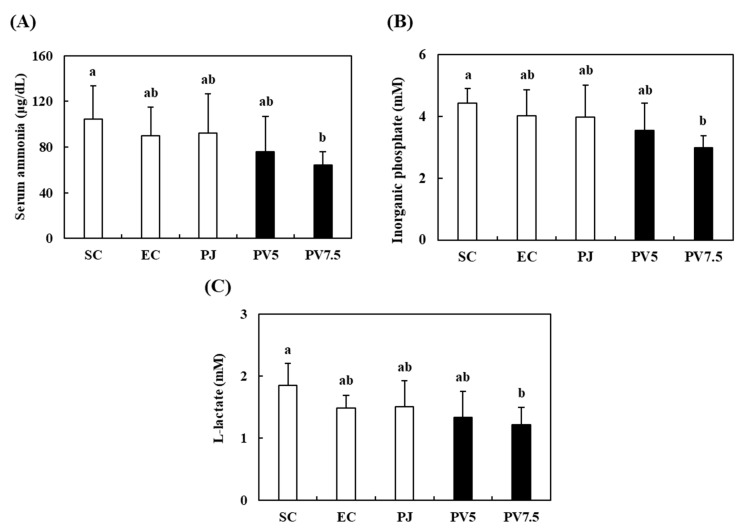
Effect of PV on serum (**A**) ammonia, (**B**) inorganic phosphorus, and (**C**) lactate in exhausted rats. Data values are expressed as the means ± S.E. (*n* = 6). SC: sedentary control, EC: exercised control, PJ: *Prunus mume* juice, PV5: 5% *Prunus mume* vinegar drink, PV7.5: 7.5% *Prunus mume* vinegar drink. Different letters on the bar are significantly different (*p* < 0.05).

The occurrence of physical fatigue is connected to energy deficits while exercising. Because large amounts of energy, fluids and amino acids are consumed during high-intensity exercise, sports drinks can help maintain the balance of fluids and resynthesis of proteins [29]. For this reason, PV could be utilized as sports drink to improve fatigue induced by exercise. Moreover, intracellular acidosis induces muscle fatigue resulting from the accumulation of lactate and inorganic phosphate [12]. During intense exercise, serum biomarkers related to fatigue such as serum ammonia, inorganic phosphate and lactate accumulate cause muscle fatigue as a result of intracellular acidosis [30]. Thus, the reduction of sensibility for fatigue is related to increasing running time and decreasing fatigue biomarkers. Liver and muscle glycogen, which are well-known sources of substrate for glycolysis and energy production, act as the first defense against energy depletion [5]. Hence, glycogen is one of the indexes of fatigue. The serum ammonia, inorganic phosphate and lactate levels of PV7.5 group were 64.57 μg/mL, 2.98 mM and 1.21 mM (Figure 4A–C). These values were reduced significantly by 28.22%, 25.91% and 18.24% relative to the EC group, respectively. When compared with the serum biomarkers in EC rats, SC and PJ rats showed no significant differences. Fushimi et al. (2001) reported that vinegar supplementation significantly reduced serum lactate and ammonia after exercise to exhaustion in rats, and Stephens et al. (2008) reported that the oral administration of acetate improved the level of blood lactate in pigs [31,32]. Based on these results, the high levels of organic acids and various free amino acids in PV might have affected the regulation of serum ammonia, inorganic phosphate and lactate. Therefore, PV administration effectively exerted anti-fatigue effect by regulating fatigue-related serum biomarkers in exercise-trained rats.

### 3.6. Effects of PV on Changes of Glycogen Accumulation

**Figure 5:**
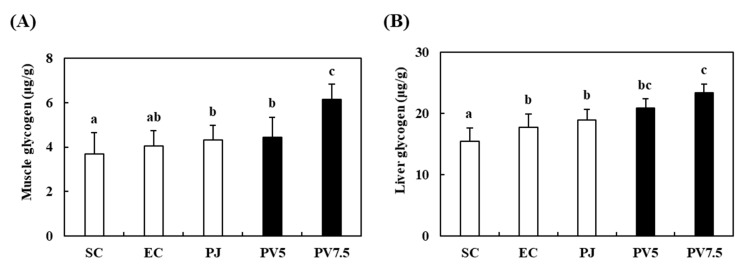
Effect of PV on glycogen accumulation of (**A**) muscle and (**B**) liver in exhausted rats. Data values are expressed as the means ± S.E. (*n* = 6). SC: sedentary control, EC: exercised control, PJ: *Prunus mume* juice, PV5: 5% *Prunus mume* vinegar drink, PV7.5: 7.5% *Prunus mume* vinegar drink. Different letters on the bar are significantly different (*p* < 0.05).

The effects of PV on liver and muscle glycogen are shown in Figure 5. The EC group showed a higher content of gastrocnemius muscle glycogen, but there were no significant differences between the SC and EC groups (Figure 5A). However, a significant elevation of glycogen content (34.25%) was observed relative to the EC and PV7.5 groups. Liver glycogen content also increased in response to supplementation with PV7.5 by up to 24.21% relative to the EC group (Figure 5B). Previous studies reported that the oral supplementation of acetic acid enhances glycogen synthesis in liver and muscle after exercise in rats and horses [10,11,31]. Therefore, this result suggests that the increase in liver and muscle glycogen levels might be related to anti-fatigue activity in high-intensity exercised rats.

### 3.7. Effects of PV on Changes of LDH and CK Activities

**Figure 6:**
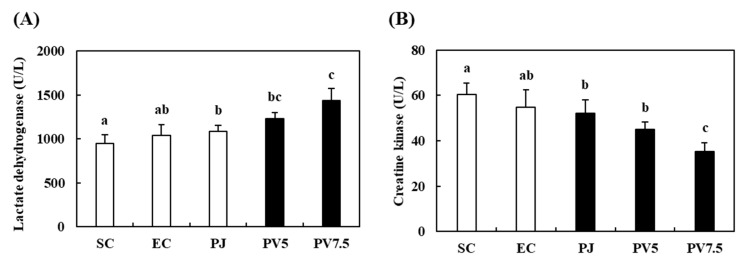
Effects of PV on (**A**) lactate dehydrogenase and (**B**) creatine kinase activities in rats exhausted by exercise. Data values are expressed as the means ± S.E. (*n* = 6). SC: sedentary control, EC: exercised control, PJ: *Prunus mume* juice, PV5: 5% *Prunus mume* vinegar drink, PV7.5: 7.5% *Prunus mume* vinegar drink. Different letters on the bar are significantly different (*p* < 0.05).

Lactate dehydrogenase (LDH) is an oxidoreductase in glycolysis that catalyzes the reversible conversion of lactic acid to pyruvate [33]. Serum creatine kinase (CK) is an important enzyme indicating muscle injury [34]. Thus, we evaluated muscle LDH and serum CK levels to evaluate the level of muscle damage. The gastrocnemius LDH level of the EC rats did not differ significantly when compared with the SC group (Figure 6A). The LDH activity of rats administered with PV7.5 significantly increased by 27.75% as compared with the EC group. As shown in Figure 6B, the serum CK level of the SC group was 60.35 U/L. The CK value of the EC group was 54.71 U/L, which was not significantly different from the comparison of the SC group. However, PV7.5 supplementation significantly reduced CK levels by 35.66% in comparison with the EC rats. In similar studies, *Prunus mume* extract improved fatigue recovery via increase in LDH activity and regulation of serum biomarkers in trained rats, and increased serum CK in response to muscle damage caused by muscle tightness, inducing fatigue [4,35]. These findings suggest that PV administration prevented fatigue by promoting the metabolism of lactic acid in muscle cells and reducing muscle damage by decreasing the level of serum fatigue markers in rats.

### 3.8. Effects of PV on Changes in MDA Level and GPx Activity in the Liver

**Figure 7:**
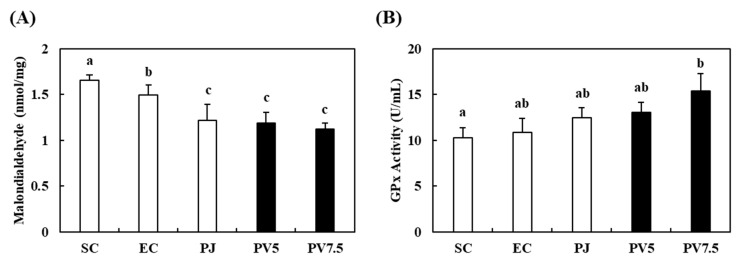
Effects of PV on (**A**) malondialdehyde and (**B**) glutathione peroxidase activities in rats exhausted by exercise. Data values are expressed as the means ± S.E. (*n* = 6). SC: sedentary control, EC: exercised control, PJ: *Prunus mume* juice, PV5: 5% *Prunus mume* vinegar drink, PV7.5: 7.5% *Prunus mume* vinegar drink. Different letters on the bar are significantly different (*p* < 0.05).

Muscle injury causes changes in the activity of antioxidant enzymes and MDA levels [34]. MDA is one of the byproducts of lipid peroxidation induced by oxidative stress. To observe changes in antioxidant enzyme and lipid peroxidation, we measured MDA and GPx levels in liver tissue by the administration of PV to exhausted exercise rats. The results revealed significant changes in response to the administration of PV. Specifically, the MDA content of the EC group decreased by 10% relative to the SC group (Figure 7A). The administration of PJ, PV5 and PV7.5 elicited decrease in MDA content of 18.35%, 20.36% and 25.05%, respectively, relative to the EC group. Administration of PV under intense exhausted exercise induced significant increases in GPx activity (Figure 7B). In the liver, PV7.5 treatment significantly increased GPx activity by 19.65% and 41.14%, although PV5 showed no difference compared to the EC rats. In previous studies, the exogenous supplementation of antioxidants and antioxidative diet decreased the levels of oxidative stress in athletes after exhaustive exercise [36]. The administration of antioxidants prevents muscle soreness in humans following exercise [37]. Moreover, Chinese black vinegar induced antioxidant activities via reactive oxygen species inhibition, as well as increases in SOD and CAT activities [38]. Taken together, these results suggest that the supplementation of vinegar with antioxidant activity enhances fatigue recovery. Therefore, the anti-fatigue activities of PV might be associated with the regulation of antioxidant enzymes in exhausted rats.

## 4. Conclusions

In this study, PV containing various free amino acids and organic acids was developed by a two-step fermentation process and evaluated by analyses of cytotoxicity and glycogen accumulation in C2C12 myoblasts as well as *in vivo* anti-fatigue effects in exhausted rats following high-intensity exercise. High levels of glycogen accumulation were observed *in vitro*, and the administration of PV contributed to preventing fatigue by regulation of serum fatigue biomarkers and muscle injury markers in exhausted rats. Furthermore, phenolic compounds such as protocatechuic acid, syringic acid and chlorogenic acid derivates in PV were identified. Collectively, PV might be expected to be used as a functional material against fatigue induced by high-intensity exercise.

## Figures and Tables

**Table 1:** Contents of organic acids and free amino acids in *Prunus mume* vinegar (PV).

Organic acids (mg%)	PJ	PV	Free amino acids (ppm)	PJ	PV
Acetic acid	N.D.	4034.46 ± 114.37	Aspartic acid	19.66 ± 4.19	7.56 ± 0.48
Oxalic acid	N.D.	72.76 ± 4.61	Tyrosine	1.12 ± 0.23	5.46 ± 0.16
Citric acid	969.23 ± 37.38	1530.65 ± 79.36	Phenylalanine	0.33 ± 0.16	4.43 ± 0.24
Succinic acid	N.D.	1075.51 ± 61.72	Histidine	0.23 ± 0.11	32.93 ± 3.51
Malic acid	352.83 ± 51.34	140.95 ± 10.58	Lysine	0.21 ± 0.14	4.11 ± 0.37
Lactic acid	N.D.	390.87 ± 26.34	Arginine	N.D.	20.76 ± 1.39
Total organic acids	1,321.06 ± 73.45	7,259.24 ± 211.93	Total free amino acid	42.78 ± 8.55	83.32 ± 5.62

Data values are expressed as the means ± S.E. (*n* = 3). N.D: not detected.

## Supplementary Materials

The following are available online at https://www.mdpi.com/2072-6643/12/5/1205/s1, Figure S1: Production of PV by two-step fermentation.
