# Supplementary material for: Anti-Fatigue Effect of Prunus Mume Vinegar in High-Intensity Exercised Rats
Source: Nutrients. 2020 Apr 25;12(5):1205. doi: 10.3390/nu12051205 (PMC7281981; doi:10.3390/nu12051205)
Supplement: Supplementary file 1 [file nutrients-12-01205-s001.pdf]

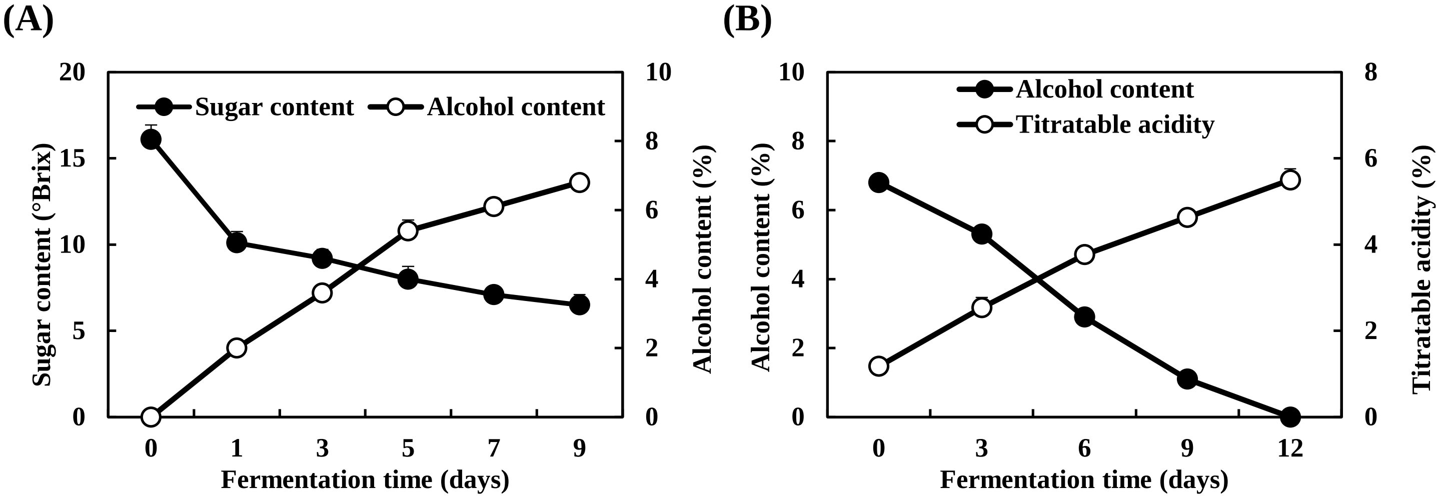

Figure S1. Production of PV by two-step fermentation. (A) Changes in sugar and alcohol content during alcohol fermentation. (B) Changes in alcohol content and titratable acidity during acetic acid fermentation. Data values are expressed as the means ± S.E. (n=3).
